# scAnnotatR: framework to accurately classify cell types in single-cell RNA-sequencing data

**DOI:** 10.1186/s12859-022-04574-5

**Published:** 2022-01-17

**Authors:** Vy Nguyen, Johannes Griss

**Affiliations:** Department of Dermatology, Medical University of Vienna, Währinger Gürtel 18-20, 1090 Vienna, Austria

**Keywords:** scAnnotatR, Cell classification, scRNAseq, Machine learning, SVM, R, Bioconductor

## Abstract

**Background:**

Automatic cell type identification is essential to alleviate a key bottleneck in scRNA-seq data analysis. While most existing classification tools show good sensitivity and specificity, they often fail to adequately not-classify cells that are missing in the used reference. Additionally, many tools do not scale to the continuously increasing size of current scRNA-seq datasets. Therefore, additional tools are needed to solve these challenges.

**Results:**

scAnnotatR is a novel R package that provides a complete framework to classify cells in scRNA-seq datasets using pre-trained classifiers. It supports both Seurat and Bioconductor’s SingleCellExperiment and is thereby compatible with the vast majority of R-based analysis workflows. scAnnotatR uses hierarchically organised SVMs to distinguish a specific cell type versus all others. It shows comparable or even superior accuracy, sensitivity and specificity compared to existing tools while being able to not-classify unknown cell types. Moreover, scAnnotatR is the only of the best performing tools able to process datasets containing more than 600,000 cells.

**Conclusions:**

scAnnotatR is freely available on GitHub (https://github.com/grisslab/scAnnotatR) and through Bioconductor (from version 3.14). It is consistently among the best performing tools in terms of classification accuracy while scaling to the largest datasets.

**Supplementary Information:**

The online version contains supplementary material available at 10.1186/s12859-022-04574-5.

## Background

Single-cell RNA-sequencing (scRNA-seq) has become a key tool for biomedical research. One of the main steps in analyzing scRNA-seq data is to classify the observed cell types.

The most common approach to annotate cell types is using cell clustering and canonical cell type-specific marker genes. However, this has several major drawbacks. First, it requires profound knowledge of a wide range of cell populations. The situation becomes more complicated if a dataset contains highly similar cell types such as T cells, ILC, and NK cells. Second, cell clusters may not be “pure” but may contain mixtures of multiple cell types. Such cases are often missed when only focusing on cluster-specific marker genes. Finally, this manual approach does not efficiently scale to large-scale studies or data reanalysis and is inherently hard to reproduce. Therefore, automated methods are needed to identify cell types in scRNA-seq data.

In recent years, several computational methods were developed to automatically identify cells. This includes methods that identify cell types by projecting cells to cell type landmarks, then inferring unknown cells close to already known cell types in the embedded space (northstar [1], scmap [2], MARS [3]). A further approach is to correlate gene expression in annotated groups/clusters of cells with unannotated populations (scCATCH [4], SingleR [5], CIPR [6], clustifyr [7], scMatch [8]). Without using annotated datasets, DigitalCellSorter [9] classifies cells based on the expression of high impact biomarkers, where the impact of the biomarkers depends on their unicity to particular cell types. A large number of algorithms use machine learning (CellAssign [10], SciBet [11], Garnett [12], CHETAH [13], SCINA [14], scPred [15], scID [16], scClassify [17]), or neural networks (ACTINN [18], MARS [3]) to automatically learn mapping functions from gene expression of annotated cells to classes of those cells. Despite this large number of cell classification approaches, several approaches show weaknesses that prevent their easy implementation into existing workflows.

We classified existing tools based on key features that we feel are required to automatically classify cell types (Table 1). Classifying individual cells instead of whole clusters can be used to cross-validate the clustering results. We only identified four tools that report ambiguous cell type assignments: MARS, DigitalCellSorter, scClassify and CHETAH. This is crucial since many cell types are closely related, such as monocytes, macrophages, and dendritic cells, which can easily lead to incorrect classification results. scClassify, CHETAH, and Garnett are the few tools classifying cells based on a hierarchical structure. This concept was also proposed by Alquicira-Hernandez et al., although it is not implemented in scPred. Hierarchical classification has the advantage that models can use features that are well suited to differentiate closely related subtypes, but might not be ideal to classify the whole lineage. Additionally, several tools are unable to not-classify cells missing in the used reference (unknown population detection). Finally, many tools fail to process large datasets as they convert the sparse matrix (where “0” values do not require memory) into a full matrix during the classification process (scClassify, SCINA, scmap-cell, and scmap-cluster). CHETAH was the only R based tool we evaluated that contains all features which we feel are necessary for accurate cell type classification. However, the confidence score reported by CHETAH is challenging to interpret and the prediction output as ‘nodes' is inconvenient for further automated analyses. Therefore, we found no R-based tool that fulfills all our criteria.

**Table 1:** Structured list of existing tools to automatically classify cell types in scRNA-seq datasets

Tools	Usage language	Level of assignment	Reference source	Prediction score	Ambiguous assignment	Unknown population detection
northstar	Python	Cluster	Dataset	No info	No info	Yes
scmap	R	Both	Dataset	Yes	No	Yes
MARS	Python	Cell	Dataset	Yes	Yes	Yes
scCATCH	R	Cluster	Database	Yes	Yes	No
SingleR	R	Cell	Dataset	Yes	No	Yes
CIPR	R	Cluster	Dataset	Yes	No	Yes
clustifyr	R	Both	Dataset	Yes	No	Yes
scMatch	Python	Cell	Dataset	Yes	No	No info
DigitalCellSorter	Python	Cluster	Markers	Yes	Yes	Yes
CellAssign	R	Cell	Markers	Yes	No	Yes
SciBet	R	Cell	Dataset	Yes, but with additional process	No	Yes, but with additional process
Garnett	R	Cell	Markers and datasets	No	No	Yes
CHETAH	R	Cell	Dataset	Yes	Yes	Yes
SCINA	R	Cell	Markers	Yes	No	Yes
scPred	R	Cell	Dataset	Yes	No	Yes
scID	R	Cell	Dataset	Yes	No	Yes
scClassify	R	Cell	Dataset	No	Yes	Yes
ACTINN	Python	Cell	Dataset	Yes	No	No
Superscan	Python	Cell	Dataset	Yes	No	Yes

Here we present scAnnotatR, a novel R/Bioconductor package to automatically classify cells in scRNA-seq datasets. scAnnotatR ships with predefined models for several cell types that can easily be extended by the user. The package uses support vector machines (SVMs) classifiers organised in a tree-like structure to improve the classification of closely related cell types. Most importantly, scAnnotatR reports classification probabilities for every cell type as well as ambiguous classification results. Therefore, scAnnotatR fills an important need in the automatic classification of cell types in scRNA-seq experiments.

### Implementation

**Fig. 1:**
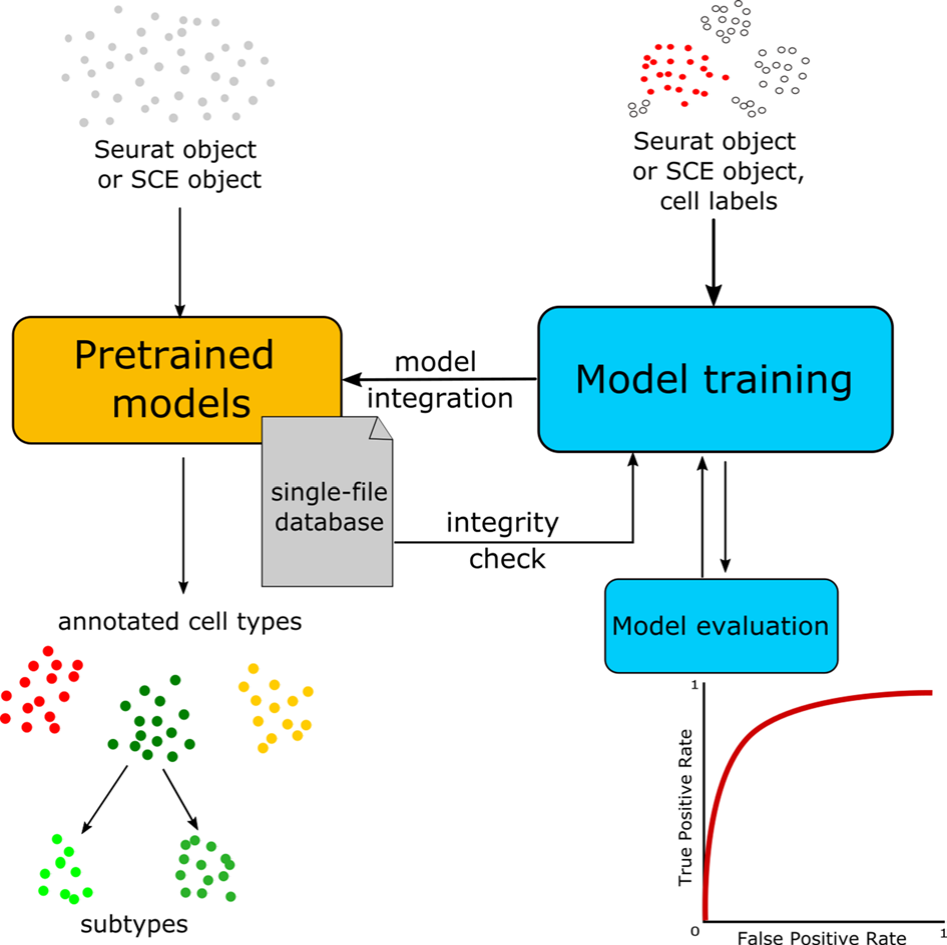
Functional structure of the package. The package supports two basic pipelines: (1) classifying cells using pre-trained models, and (2) training new classification models to extend the existing classifier database. These models can be automatically evaluated using dedicated in-built functions

scAnnotatR is an R Bioconductor package to classify cell types using pre-trained classifiers in scRNA-seq datasets. The package revolves around an S4 class called scAnnotatR. Each object of the class defines a classifier of a cell type wrapping 5 pieces of information: the classified cell type corresponding to the name of the classifier, a support vector machine (SVM)-based model returned by the caret package [19], a feature set on which the model was trained, a prediction probability threshold and the parent of the classified cell type (if available). Trained models are stored in a named list which are referred to as a classifier database. The package ships with built-in classifiers which can easily be extended or even replaced by the user (Fig. 1).

Similar to Abdelaal et al. [20], we found that linear kernels outperform other more sophisticated kernels throughout our initial benchmark (Additional file 1: Fig. S1). To support the rejection of unknown cell types, one classification model is responsible for one cell type, identifying cells belonging to a specific cell type versus all other cells. Classifiers are stored in a hierarchical tree-based structure allowing the definition of “parent” and “child” classifiers. In such cases, cells are first classified using the parent classifier. Only cells identified as that specific cell type are then further classified using the respective child classifier.

scAnnotatR is compatible with both Seurat [21] and Bioconductor’s SingleCellExperiment [22] object. It ships with pre-trained models for most basic immune cells. Therefore, it can easily be integrated into the vast majority of existing scRNA-seq workflows.

Finally, scAnnotatR offers a user-friendly environment to train and test new cell classifiers. All functional parameters are adjustable and configurable, which gives the user full control during the training process. Thereby, scAnnotatR offers a complete framework for the automatic classification of cell types in scRNA-seq datasets.

## Results

### Hierarchical classification models help identify unrecognized sub-populations

A key challenge in the characterisation of cell types in scRNA-seq datasets is to what level of detail cell types should be classified. Several research questions focus on very specific subtypes, for example specific B cell phenotypes. At the same time, other B cell subtypes may be of less interest—or be unexpected at all. In tools that do not support hierarchical classification models researchers have to either classify all B cells at the same level of detail (with the danger of missing rare subtypes) or leave a large portion of cells unclassified.

**Fig. 2:**
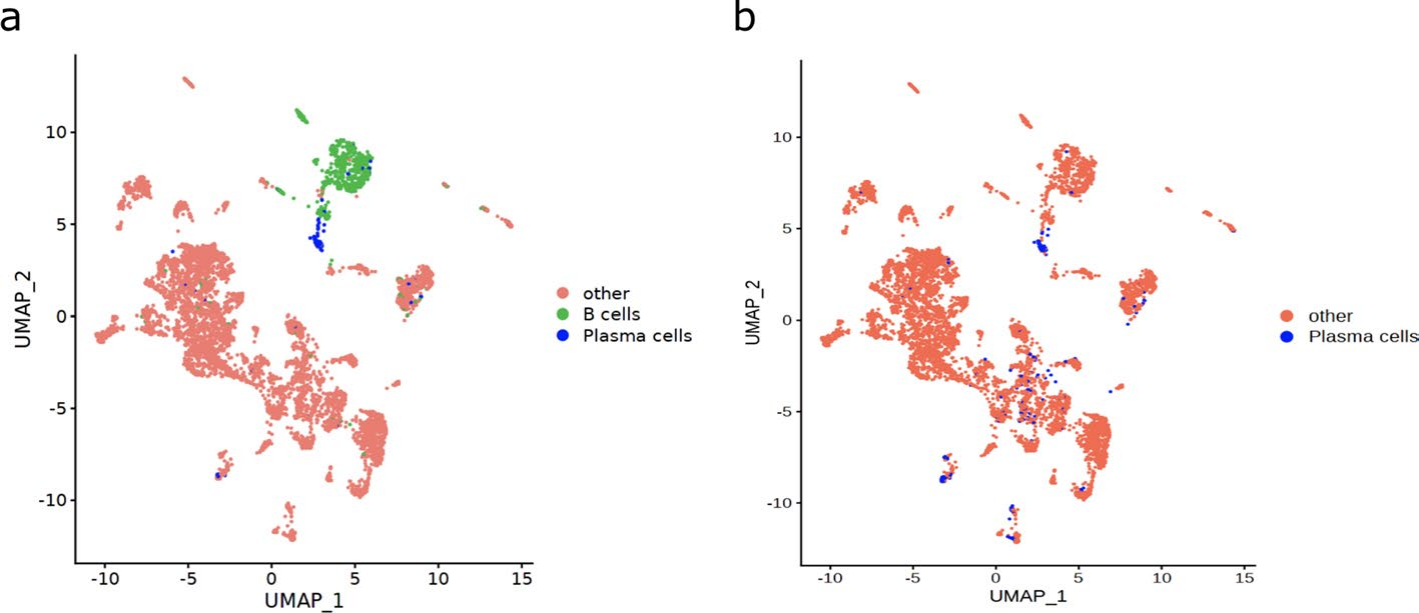
UMAP plot showing the classification results in the Jerby-Arnon melanoma dataset. **a** Classification results of a plasma cell classifier as a child of the more general B cell classifier. **b** Classification results of a plasma cell classifier trained separately without any parent classifier

scAnnotatR’s hierarchical organisation of cell classification models is ideally suited for such targeted cell classification approaches. First, researchers can train a parent classifier to identify all cells belonging to the general cell type of interest. In a second step, they can now create a child classifier(s) to focus on their subtype(s) of interest. Figure 2 highlights these two approaches. scAnnotatR’s inbuilt classifiers contain a hierarchical model for overall “B cells” and its child terminally differentiated “plasma cells”. Figure 2a highlights that the dataset contains several plasma cells, but a large portion of the general “B cells” is only captured by the parent classifier. More importantly, a group of cancer-associated fibroblasts (lower left group of cells, Fig. 2b) were misclassified as plasma cells. These express SDC1, a sensitive but not specific plasma cell marker. Due to their additional expression of FAP, PDGFRA, PDGFRB, TAGLN, and COL1A1 we can be certain that they are not plasma cells. The general B cell classifier was able to correctly distinguish these cells (Fig. 2a). This example highlights how the hierarchical structure of classifiers can increase classification accuracy.

### scAnnotatR outperforms existing tools in terms of accuracy, sensitivity, and the detection of unknown cell types

We compared scAnnotatR’s performance against existing tools using two benchmarks: first, a group of datasets containing discrete cell populations and second a group with closely related immune cell populations. The benchmarks included ten other existing tools. SingleR [5] selects the most variable genes for each cell type in an annotated dataset. Then, cell types are identified in an unlabelled dataset by correlating the expression values. CHETAH [13] selects the top differentially expressed genes (DEG) and finds the distribution of correlation between cells in each cell type; unknown cells are then classified by the high cumulative density of a cell type correlation distribution. scmap-cluster and scmap-cell calculates the cosine similarity and Pearson and Spearman correlations between the unidentified cells and the reference cell/clusters to infer the closest populations. scClassify uses a combination of feature selection methods (mainly limma) to train one or multiple classifiers, then uses one or multiple classifiers to classify cells and has those classifiers vote for cell identification. SciBet [11] retrieves cell type markers and eliminates noisy genes using the E-test. For each cell type, SciBet learns a multinomial model to form a likelihood function defining the probability of each cell to belong to a cell type, hence cell annotation relies on a likelihood maximization process. Garnett [12] requires a list of marker genes as input to choose a set of representative cells and train multinomial classifiers. clustifyr [7] is the only tool working on the cluster level. It identifies cell types through the correlation of cluster gene expression with annotated cell expression values. SCINA [14] relies on user-supplied marker genes. It uses a bimodal distribution to identify cells where the marker genes are higher or lower expressed. It then uses the Expectation–Maximization (EM) algorithm to calculate the likelihood of one cell belonging to a cell type. Similar to scAnnotatR, scPred [15] trains machine learning models for each cell type in the reference dataset, with two differences: 1- principal components are used as features in the machine learning models instead of individual genes and 2- classification models are not organised in a hierarchical structure. This explains the difference in performance between scPred and scAnnotatR. This wide collection of existing tools ensures that we arrive at a comprehensive assessment of scAnnotatR’s performance in comparison to the state-of-the-art.

The benchmark evaluates the accuracy calculated on the dataset level, and the average sensitivity, and specificity across all cell types in the tested datasets. Additionally, the ability to correctly deal with unknown cells is a key aspect in the automatic classification of cell types. Reference datasets may always be incomplete. Therefore, tools need to be able to recognize such unknown cells to avoid a misinterpretation of the data. To assess this ability, we calculated the unknown population detection rate, which is defined as the number of correctly unassigned cells over the total number of cells that are not present in the reference. Thereby, we arrive at a comprehensive overview of the tools’ performance.

#### Classifying discrete cell populations

We performed the benchmark using a sixfold cross-validation scheme with six pancreas datasets [23–27] (Additional file 1: Fig. S2). In each fold, one of the six datasets was used for training, the other five for testing. scAnnotatR, CHETAH, and scClassify are the only evaluated tools able to return ambiguous/intermediate cell type assignments. In order to ensure a fair comparison, the accuracy, sensitivity and specificity was calculated using two methods for these tools. Once, a correct intermediate assignment was accepted as correct classification (marked with a star (*) after the name). In the second approach only unambiguous classifications were counted as correct (see Additional file 1 for details). This sixfold cross-validation benchmark thereby ensures that we arrive at an accurate and comparable estimate of each tool’s performance.

**Fig. 3:**
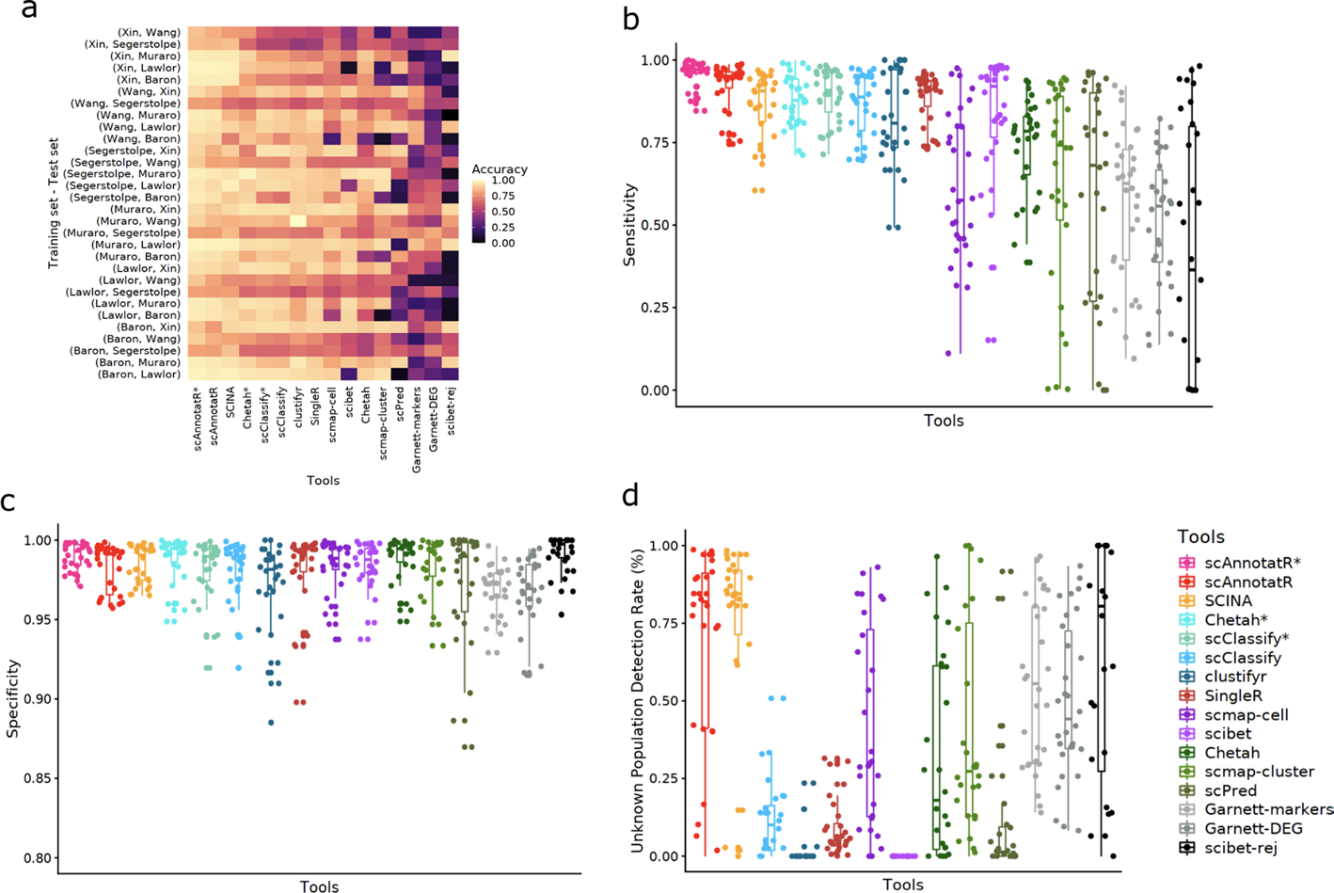
Benchmark results when classifying distinct cell populations in six pancreas datasets. The benchmark was performed in a sixfold cross-validation scheme where one dataset was used for training and the other five for testing. All metrics were ordered by the tools’ mean accuracy. (*) indicates results where ambiguous classifications were accepted as correct. Garnett-markers and Garnett-DEG are two evaluations of Garnett, one using the same markers as scAnnotatR (Garnett-markers) and one using top 10 DEG (Garnett-DEG). scibet-rej evaluates SciBet’s performance using a confidence threshold of 0.4. **a** The prediction accuracy in each training and test set for all tools. **b** Average sensitivity and **c** specificity as the mean sensitivity and specificity, respectively, across all classified cell types for each dataset and iteration. **d** Proportion of cells not present in the training data, that were correctly not-classified

Throughout all iterations, scAnnotatR was consistently among the tools with the highest accuracy, sensitivity, and specificity (Fig. 3a–c). As expected, accepting ambiguous results as correct improved the performance of the respective tools. Only scAnnotatR and SCINA were able to reach a good accuracy, sensitivity, and specificity while being able to correctly not-classify unknown cell types (Fig. 3a, b, d). While SingleR, scClassify, SciBet (default settings), and clustifyr, showed high accuracy and sensitivity, they failed in not-classifying unknown cells (Fig. 3d). The increased detection of unknown cells by scmap-cell, scmap-cluster, Garnett, and scibet-rej comes at the cost of reduced sensitivity and accuracy. scPred’s comparably low performance may be due to the fact that the training and testing datasets were processed using different workflows (Fig. 3a–d). Its default RBF training kernels seemed to overfit the data. Nevertheless, even when processing all datasets using the same workflow and manually optimising the used kernels, scPred’s performed worse than scAnnotatR (Additional file 1: Fig. S3). Meanwhile, that fact that clustifyr works on the cluster level leads to a win all or lose all scenario. In datasets where the clustering results were suboptimal, clustifyr’s performance decreases dramatically. Altogether, scAnnotatR showed the highest accuracy while still being able to not classify unknown cells.

#### Closely related populations

Our second benchmark tested the differentiation of closely related immune cell types. The benchmark was performed in a multiple-fold cross-validation scheme using two levels of cell annotations with ten annotated datasets: the PBMC 3 k dataset as analyzed in the Seurat v3.1 tutorial [28], the PBMC 500 dataset analyzed by ILoReg v1.0 tutorial [29], the seven subsets in the PBMC dataset by Ding et al. [30], and the SCP345 PBMC dataset [31]. The SCP345 PBMC dataset could not be included in the second level annotation benchmark as it lacks detailed cell annotations. This selection of datasets ensures that we can assess the classification performance in closely related cell types.

In the first level of cell annotations, scAnnotatR used the same markers as the in-built classifiers for T cells, B cells, NK cells, monocytes (macrophages), and dendritic cells to train the classifiers on the corresponding training set. In the second level of the benchmark, scAnnotatR used additional markers from the in-built classifiers for CD4+ T cells, CD8+ T cells, CD14+ monocytes and CD16+ monocytes. Garnett and SCINA used the top 10 differentially expressed genes of each cell type in the corresponding training set. For Garnett and scAnnotatR, the hierarchical structure of cell types is a predefined input.

Additionally, we assessed the influence of an adapted prediction threshold for scAnntotaR. Next to the trained model, scAnnotatR’s classifiers use a prediction threshold before classifying a cell. This threshold can be adapted without retraining the classifiers. Therefore, we once evaluated scAnntotatR with its default thresholds of 0.5, and once with adapted thresholds to increase sensitivity. This highlights how scAnnotatR can easily be optimised for different use cases.

**Fig. 4:**
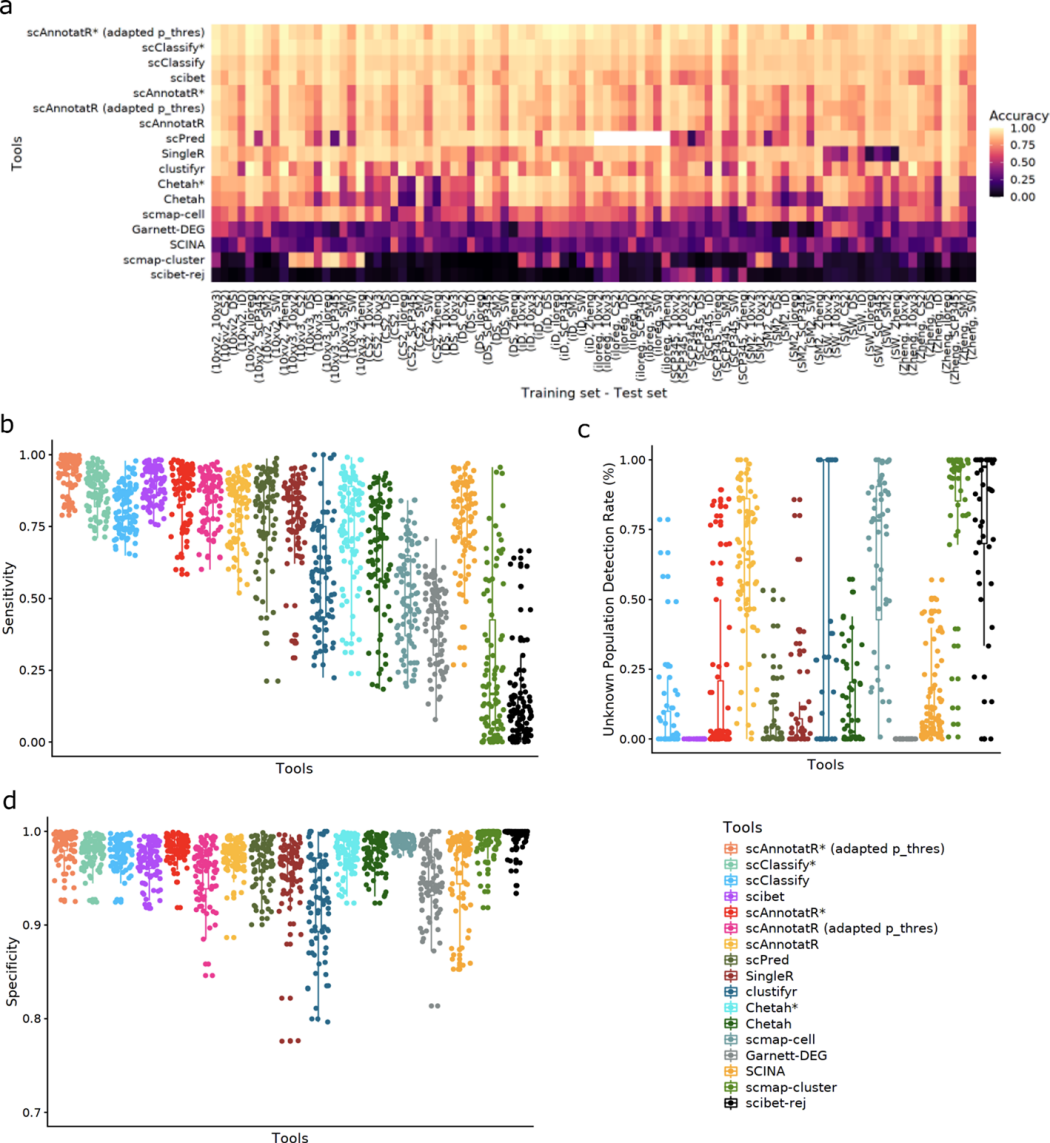
Benchmark evaluating the classification accuracy for closely related immune cell types in ten datasets using the more general (level 1) annotations. The benchmark was performed in a tenfold cross-validation scheme where one dataset was used for training and the other five for testing. All metrics were ordered by the tools’ mean accuracy. (*) indicates results where intermediate/ambiguous classifications were accepted. scibet-rej represents SciBet with a confidence threshold of 0.4. Blank areas indicate comparisons that could not be performed with the respective tool. **a** The prediction accuracy in each training set and test set for all tools. Panels show the sensitivity (**b**), specificity (**d**). The shown values (points) represent the mean sensitivity and specificity, respectively, across all classified cell types across the five evaluated datasets per iteration. Panel (**c**) shows the proportion of cells not present in the training data, that were correctly not-classified

**Fig. 5:**
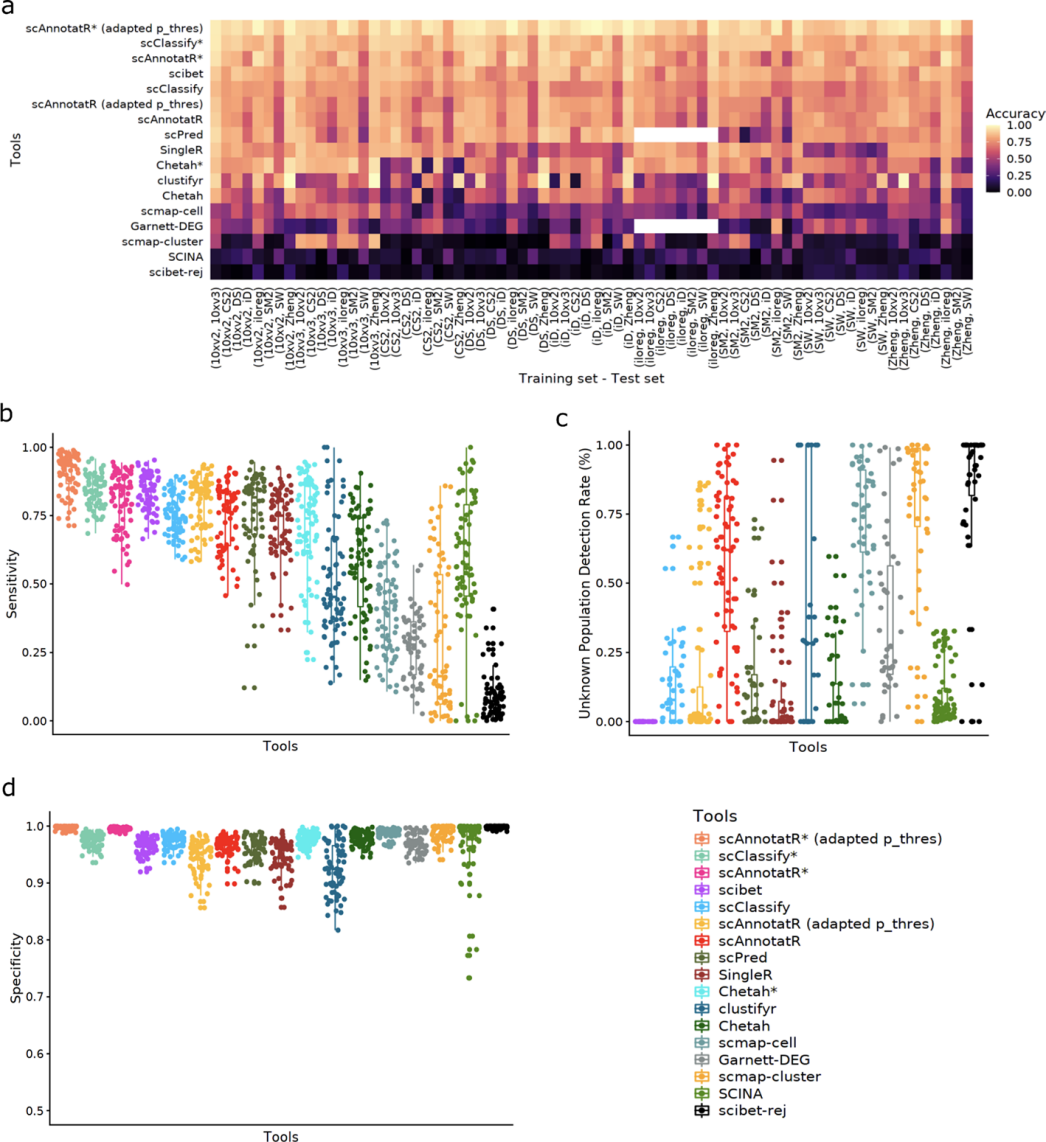
Benchmark evaluating the classification accuracy for closely related immune cell types in nine datasets using the detailed cell annotations (level 2). The benchmark was performed in a ninefold cross-validation scheme where one dataset was used for training and the other eight for testing. All metrics were ordered by the tools’ mean accuracy. (*) indicates results where intermediate/ambiguous classifications were accepted. scibet-rej represents SciBet with a confidence threshold of 0.4. Blank areas indicate comparisons that could not be performed with the respective tool. **a** The prediction accuracy in each training set and test set for all tools. Panels show the sensitivity (**b**), specificity (**d**). The shown values (points) represent the mean sensitivity and specificity, respectively, across all classified cell types across the five evaluated datasets per iteration. Panel (**c**) shows the proportion of cells not present in the training data, that were correctly not-classified

In general, the performance of all tested tools was worse when classifying closely related cell types (Fig. 4). The ranking of tools did not change dramatically, except for scPred which increased its rank to the middle-top (Fig. 4). Optimising scPred’s classifiers again improved its performance, which was still lower than scAnnotatoR’s (Additional file 1: Fig. S3). scAnnotatR was again the only top-performing tool with an acceptable unknown population detection rate. scClassify, SingleR, SciBet, scPred, and SCINA had high accuracy and/or sensitivity but low unknown population detection rates, while clustifyr, scmap-cell, scmap-cluster, and Garnett had a higher unknown population at the cost of decreased sensitivity. When using the more detailed cell annotations both scibet’s, scPred's and scClassify’s performance decreased compared to scAnnotatR (Fig. 5). Compared to the previous benchmark, SCINA's ability to not classify unknown cells decreased. As SCINA relies on lists of positive-only marker genes, closely related cell types are more difficult to distinguish. In both levels of cell annotations, the performance of clustifyr was highly variable, depending on the clustering results. As expected, the classification tools supporting ambiguous cell identification (scAnnotatR, CHETAH, scClassify) generally had better performance in the intermediate assignment-accepted scenario than in the normal one. Overall, scAnnotatoR showed a consistently good performance when classifying closely related immune cell types.

### scAnnotatR scales to large datasets

**Fig. 6:**
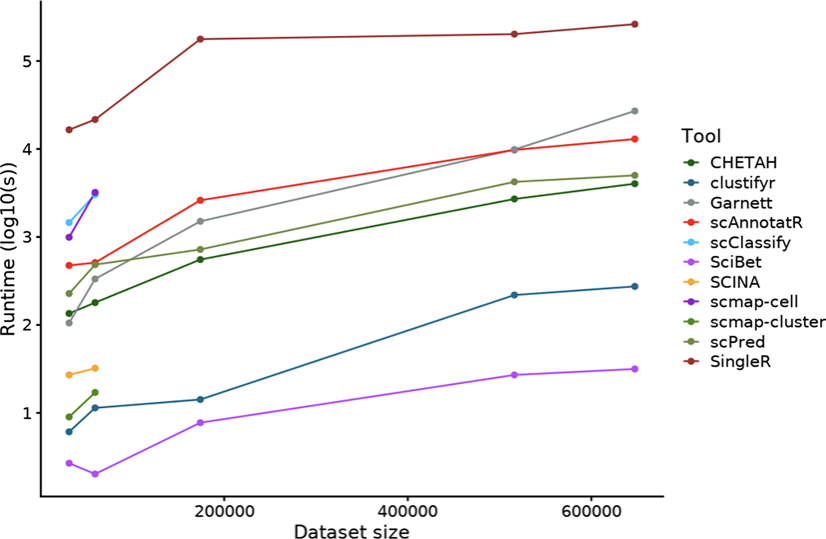
Prediction runtime of all tools measured over five large datasets

We evaluated the scalability of all applications on five large datasets (Fig. 6). SCINA, scClassify, and scmap (both versions) were unable to process the largest studies. These tools convert the sparse matrix into a full expression matrix which dramatically increases memory usage. Even though our machine was equipped with 200 GB of RAM, this was not enough. SciBet and clustifyr were the fastest evaluated tools, followed by CHETAH, Garnett, scPred and scAnnotatR who processed the two largest datasets in hours. SingleR was significantly slower than the other tools and needed several days to process the three largest datasets. Among the top performing tools, scAnnotatR was the only one able to process the largest datasets in a reasonable amount of time.

## Discussion

Automatic cell type identification in scRNA-seq datasets has become a highly active field and is an essential method to alleviate a key bottleneck in scRNA-seq data analysis. Our benchmarks showed that many of the available tools experience a tradeoff between the accuracy/sensitivity and the unknown population detection rate. In our benchmarks, scAnnotatR, scClassify, and SingleR were able to consistently achieve a high accuracy and sensitivity in both benchmarks, but only scAnnotatR was able to accurately detect unknown populations. Finally, a surprisingly large number of well performing tools were unable to process increasingly common large datasets. Overall, scAnnotatR was the only assessed tool with consistently high classification performance able to process large datasets.

A large group of algorithms, such as MARS [3] or SingleR [5], rely on a single annotated reference dataset. In our experience, this approach is often limited since a single dataset may not contain all cell types of interest. When multiple datasets have to be merged, data size and computationally cost quickly increase dramatically as shown for SingleR in our benchmark. Additionally, sharing annotated reference datasets is complicated by their size. The advantage of scAnnotatR and other related tools is that the cell type’s properties are learned from a reference dataset, but the reference dataset is no longer necessary to apply the model. This makes the learned models easily transferable, shareable, and reproducible as highlighted by the models shipped as part of scAnnotatR.

scAnnotatR was developed to offer full control and detailed information related to the cell annotation. Additionally, it is among the few tools to provide a dedicated infrastructure to train new cell classifiers. It is impossible to create references that suit all experimental designs. We explicitly provide functions that greatly simplify the training and, most importantly, evaluation of new cell types. Plans are under way to support a GitHub-based central repository for cell type classifiers that also supports multiple species. This will help researchers to quickly share their own classifiers. scAnnotatR therefore is a scalable, accurate and reproducible method to automatically classify cell types in scRNA-seq datasets.

## Conclusions

scAnnotatR is among the most accurate and scalable methods to classify cells in scRNA-seq datasets. Most importantly, it is able to correctly not-classify unknown cell types. scAnnotatR provides a complete framework to train, test, and store new classifiers and is compatible with both Seurat and Bioconductor’s SingleCellExperiment class. Thereby, it can be quickly incorporated in virtually all R-based scRNA-seq workflows.

### Availability and requirements

- Project name: scAnnotatR.
- Project home page: https://github.com/grisslab/scAnnotatR.
- Operating system(s): Platform independent.
- Programming language: R.
- Other requirements: R packages (dplyr, ggplot2, caret, ROCR, pROC, data.tree, methods, stats, e1071, ape, kernlab, utils, AnnotationHub).
- License: MIT + file LICENSE.
- Any restrictions to use by non-academics: no.

## Supplementary Information

**Additional file 1.** Supplementary methods and figures.

## Data Availability

scAnnotatR is freely available as open source software on GitHub at https://github.com/grisslab/scAnnotatR and through Bioconductor since version 3.14 at https://bioconductor.org/packages/release/bioc/html/scAnnotatR.html. The complete code to perform and analyse the benchmarks and to create all figures is available on CodeOcean at https://doi.org/10.24433/CO.8414972.v1.

## Abbreviations

scRNA-seq: Single cell RNA sequencing
WKNN: Weighted k-nearest neighbors
KNN: k-nearest neighbors
SCE: SingleCellExperiment
NK: Natural killers
DC: Dendritic cells
pDC: Plasmacytoid dendritic cells
DEG: Differentially expressed genes
PBMC: Peripheral blood mononuclear cell
HIV: Human immunodeficiency virus
SVM: Support vector machine
HCA: The Human Cell Atlas
UMAP: Uniform Manifold Approximation and Projection
