# Supplementary material for: scAnnotatR: framework to accurately classify cell types in single-cell RNA-sequencing data
Source: BMC Bioinformatics. 2022 Jan 17;23:44. doi: 10.1186/s12859-022-04574-5 (PMC8762856; doi:10.1186/s12859-022-04574-5)
Supplement: Supplementary file 1 — Additional file 1. Supplementary methods and figures. [file 12859_2022_4574_MOESM1_ESM.pdf]

scAnnotatR: Framework to accurately classify cell types in single-cell RNA-sequencing data - Supplementary Data

Vy Nguyen[1], Johannes Griss[1]

[1] Department of Dermatology, Medical University of Vienna, 1090 Vienna, Austria

Table of contents

Supplementary Methods **2**

Classification process 2

Training new classifier process 2

Datasets 4

Data preprocessing and cell type assignment 4

Pretrained learning models for basic immune cells 8

Benchmarking discrete populations using the pancreas datasets 8

Benchmarking closely related populations 10

Runtime estimation 11

Supplementary Figure 1 **13**

Supplementary Figure 2 **14**

Supplementary Figure 3 **15**

References **17**

Supplementary Methods

The complete code to 1) prepare the test datasets, 2) run all tools, 3) evaluate the benchmark results, and 4) create all benchmark figures of the manuscript is available on CodeOcean at https://doi.org/10.24433/CO.8414972.v1.

Classification process

The core classification task is performed using linear SVM-based learning models on predefined sets of features through the caret R package. C was constantly set at 1. Cross-validation and down-sampling was controlled by caret while training.

scAnnotatR further supports the concept of "child" and "parent" classifiers. Child classifiers are trained and applied only to cells that were already classified by the respective parent classifier. Internally, scAnnotatR continuously ensures that the classifier database is consistent and that child classifiers are only added to a database if the respective parent classifier is present. This structure can be visualized using the "visualize_tree" function. Internally, it uses the data.tree R package to store the hierarchical structure of cell types. Taking the database location provided by users, the package automatically loads the database, retrieves the classifiers, forms cell type relationships and creates a tree of cell types. The tree has nodes corresponding to main or parent cell types and leaves corresponding to cell types having no children. Thereby, the user can get a quick overview over all available cell types.

scAnnotatR supports both Seurat and Bioconductor's SingleCellExperiment class objects as input to its central "classify_cells" function. The classification result is returned as new metadata slots in the input object storing the cell type(s) and the respective classification probabilities. This includes possible ambiguous cell type assignments with all corresponding candidates, the classification probability for every cell type, as well as the most probable cell type. Thereby, the classification results can be directly visualized and further analysed using the respective standard plotting functions and workflows of the respective scRNA-seq pipeline.

Training new classifier process

scAnnotatR simplifies the task of training and evaluating new classifiers. The training process ("train_classifier" function) supports both Seurat and SingleCellExperiment objects as input and produces an scAnnotatR object. The user only has to specify which features are used for the training process. More general markers, which are expressed by a large cell population, help increase the sensitivity of the models but decrease their specificity. More specific markers help to better

distinguish the training cell types versus the others but also decrease the sensitivity of the trained models. In some cases, negative markers are also used to differentiate between closely related cell types. In low depth-sequencing data, the expression levels of marker genes may show very different distributions. To solve this issue, the train_classifier function automatically performs a z-score transformation on the input data. A balancing process ensures that an equal number of cells are present in the target and the non-target class. Finally, the caret training function is used to train the classifier. Thereby, all steps required to train a new classifier are available in a single, simple-to-use function.

The hierarchical structure of classifiers is one of the key features of scAnnotatR. This allows us to 1/ better reflect the natural biological relationship between cell types and/or between cell type and phenotypes, and 2/ increase the prediction accuracy. For instance, a CD4+ T cell must be positive to T cell classifier, and a cell negative to T cell classifier must never be a CD4+ T cell. Moreover, it enables us to select the most appropriate markers at each level. For example, marker genes helpful in differentiating T vs. all other cells are not helpful to differentiate CD4+ from CD8+ T cells. Finally, this feature may allow us to recognize unexpected sub-populations as mentioned in the manuscript (such as T cells not further classified).

The parent-child relationship of two classifiers is defined during the training process. Most commonly, this follows the developmental trajectory of cells. . There are cases though, where this is not directly possible. Here we recommend to base this decision on the most distinct markers available. For example, central memory CD4+ T cells could be defined as a child of CD4+ T cells or central memory T cells. Since it is easier to differentiate CD4+ from CD8+ T cells, we recommend first training classifiers for these cell types and then creating child classifiers to identify the final central memory CD4+ T cells.

It is worth mentioning that the efficiency of classifier hierarchical structure increases with the increasing number of mutual markers. Therefore, a fully differentiated phenotype or too distinctive subtype might not be relevant to be a child of any other cell type because it has lost the majority of markers existing in their relatives. For instance, Langerhans cells (LC) have been supposed to be a subset of macrophages[1]. However, LC markers are far different from this potential parent, since LC expresses langerin (CD207) and CD1a, and macrophages express CD14 and/or CD16. In this case, we would propose to keep LC classifier as an independent classifier from the macrophage classifier. For that reason, we leave the user an option to choose training independent classifiers besides hierarchical structured classifier.

The testing process through the “test_classifier” function follows a similar process as the training function. Using an independent test dataset as input, the test_classifer function calculates an overall AUC score, the accuracy, sensitivity and specificity of the classifier at the default probability threshold (0.5) and at thresholds from 0.1 to 0.9 with steps of 0.1 to simplify the tuning of the probability threshold. A setter for the prediction probability threshold is available to adjust the sensitivity and specificity of the trained models, based on the specific research question or particular user’s interest. Once the classification model’s performance meets the user’s expectations, scAnnotatR provides several functions to store the classifiers in a common database. These functions ensure that the database remains consistent with respect to parent and child classifiers. Thereby, scAnnotatR provides a complete infrastructure to train and evaluate new classifiers.

Datasets

We used 19 public scRNA-seq datasets for the creation of the in-built classifiers and the benchmark of the packages. For the in-built classifiers, we used 4 datasets: the Sade-Feldman *et al.* melanoma dataset (GSE120575)[2], the Jerby-Arnon *et al.* melanoma dataset (GSE115978)[3], the Reynolds *et al.* skin cell atlas [4], and the Stephenson *et al.* Covid-19 cell atlas [5]. In the pancreas benchmark, we used six pancreas datasets from Baron *et al.* (GSE84133)[6], Muraro *et al.* (GSE85241)[7], Segerstolpe *et al.* (E-MTAB-5061) [8], Wang *et al.* (GSE83139)[9], Xin *et al.* (GSE81608)[10], and Lawlor *et al.* (GSE86473) [11].

Ten PBMC datasets were used to evaluate the performance on closely related cell types: seven (Smartseq2, SeqWell, inDrop, CelSeq2, DropSeq, 10xv2, and 10xv3) from Ding *et al.* [12], the PBMC 3k from Zheng *et al.* [13], the SCP345 PBMC dataset [14], and one PBMC 500 dataset, which is a subset of Zheng's PBMC 3k processed by the ILoReg pipeline [15]. The SCP345 PBMC dataset could not be included in the second level of the benchmark as it lacks detailed immune cell type annotations.

To benchmark the scalability and performance of the tools we used five datasets: the (joint) Ding PBMC dataset, the Reynolds skin cell atlas, and the Stephenson Covid-19 cell atlas, the Zilionis lung dataset, the HIV1 dataset.

Data preprocessing and cell type assignment

scRNA-seq data was downloaded from GEO for the Sade-Feldman and the Jerby-Arnon datasets (TPM counts). For the Sade-Feldman melanoma dataset, we preprocessed the data following the

authors' approach: First, we filtered out mitochondrial genes. We then retrieved only cells expressing at least 1000 features and only features expressed in at least 3 cells. Finally, we kept only cells with housekeeping genes expressed at low levels: log2(TPM + 1) < 2.5. For the Jerby-Arnon dataset, we eliminated mitochondrial genes, and filtered out cells having less than 1000 expressed genes and genes expressed in less than 3 cells. The datasets were then normalized and scaled using the basic pipeline of Seurat v3 regressing out confounders, such as patients (for Sade-Feldman dataset), samples and cohorts (for Jerby-Arnon dataset). After that, the data dimension was reduced to the first 40 (Sade-Feldman dataset) and 45 (Jerby-Arnon dataset) principal components. clustering was performed with default parameters. Cell annotation in these datasets was manually assigned on the cluster-level based on known canonical markers (Table 1).

Five pancreas scRNA-seq datasets (Baron, Muraro, Segerstolpe, Wang and Xin) were preprocessed based on the protocol proposed by the Hemberg lab [16]. The datasets were then normalized and scaled following v3 Seurat SCTransform protocol with regressing out main confounders: samples, patients/donors, diseases/conditions, and batches. Number of principal components for the five datasets (Baron, Muraro, Segerstolpe, Wang and Xin) are 45, 40, 45, 40, and 30, respectively. Nearest neighbors and clusters were computed using the default parameters, except clusters in Wang dataset were calculated at resolution = 1.

Cells in Baron and Xin datasets were annotated by authors. Cell types in three other datasets were manually assigned on the cluster-level and based on known canonical markers (Table 1).

Cell types	Markers
B cell	CD19, MS4A1, CD79A, CD79B, SDC1
plasma cell	CD19, SDC1
T cell	CD3D, CD3E, CD3G, CD8A, CD8B, CD4
CD4+ T cell	CD3D, CD3E, CD3G, CD4
CD8+ T cell	CD3D, CD3E, CD3G, CD8A, CD8B
NK	CD2, NCAM1, NCR1, KLRD1
monocyte	CD14, FCGR3A, FCGR3B, CD4
dendritic cell	FCER1A, CST3

melanocyte	PMEL, MLANA, TYR
endothelial cell	CD93, CD34, LYVE1
cancer-associated fibroblast	FAP, PDGFRA, PDGFRB, TAGLN, COL1A1
alpha	GCG
beta	INS
delta	SST
gamma	PPY
epsilon	GHRL
ductal	KRT19
acinar	CPA1
endothelial	KDR, ESAM, FLT1, CDH5
mesenchymal	SERPINE1

Table 1: Markers for cell type identification in Seurat analyses

The last pancreatic dataset (Lawlor) was retrieved from the scRNAseq R package [17]. The preprocessing and analysis of this dataset was done following the pipeline proposed in the Orchestrating Single-Cell Analysis with Bioconductor book, Chapter 32 [18]. This dataset was already annotated. Two versions of this dataset were created to further benchmark scPred. A Seurat object was converted from the original SCE object with cell PCA coordinations and gene/feature loadings extracted from that SCE object. Another Seurat object of this dataset was also created following the SCTransform pipeline and principal component analysis (same as the five other pancreas datasets), because scPred is intended to be used with Seurat objects where the same normalization method was applied on the training and test data.

The PBMC 3k was analyzed using Seurat v3.1 following the respective vignette [19]. The PBMC 500 dataset was preprocessed and analyzed according to the ILoReg v1.0 vignette [15]. The Ding's joint PBMC was downloaded from the Single Cell Portal [20]. Here, we got the normalized log counts per 10K data consisting of three files: barcodes.txt, genes.txt, and meta.data.new.txt. The whole joint dataset was loaded into a Seurat object, then split into multiple smaller Seurat objects corresponding to seven sequencing protocols. Original cell labels from the authors were used. Similar to the Lawlor pancreas dataset, we created two additional versions of the Ding *et al.* PBMC

dataset to further evaluate scPred. One version with the original normalization with PCA coordination and gene/feature loadings newly produced by applying Seurat's ScaleData and RunPCA functions. The other version was obtained by re-analyzing the dataset following the SCTransform approach and principal component analysis. The PBMC 500 dataset originally processed by ILoReg cannot be converted to Seurat object with PCA coordination and feature loadings because ILoReg does not rely on the feature-based PCA but uses probability cluster-based PCA. This dataset therefore can only be preprocessed again using the Seurat SCTransform process.

The HIV1 dataset was downloaded from the Single Cell Portal [21] and processed using Seurat's scTransform pipeline regressing out the donor identification. 50 principal components were used for clustering and the cell labels were provided by authors and integrated into our Seurat object.

The human lung dataset by Zilionis *et al*. [22] is available through the scRNAseq R package [17] in the form of a SCE object. This dataset was used only for runtime benchmark, therefore we kept as many cells as possible. Therefore, only 73 cells having no expression on all genes were eliminated from the SCE object. The following processing steps relied on the pipeline proposed by the Orchestrating Single-Cell Analysis with Bioconductor, Chapter 42 [23]. Particularly, the dataset was log normalized by the library size. We then blocked on the sample (Library in cell metadata) information to remove batch effect while selecting top 5000 highly variable genes. We used fastMNN proposed by batchelor to correct the batch effect on the reduced dimensions level. Therefore, MNN reduced dimensions were used for the UMAP generation and cell clustering. The analysis stopped after the clustering process.

The Reynolds skin cell atlas was downloaded via the Development Cell Atlas portal [24] in the H5AD format. Using the scanpy workflow, we loaded the dataset into an AnnData object, processed and analyzed the dataset based on the authors' description. We converted the resulting AnnData object (without the embedded spaces) to a SingleCellExperiment object using the anndata2ri library in python.

The SCE object of the HIV1 dataset, the joint PBMC Ding dataset, the Zilionis lung dataset, and the Reynolds skin cell atlas were converted to Seurat objects to further benchmark scPred including the original PCA information.

Lastly, the Stephenson Covid-19 cell atlas was retrieved from the COVID-19 Cell Atlas portal [25]. The data was log normalized and stored as an AnnData object, in an H5AD formatted file. Similar to the Reynolds skin cell atlas, the mentioned AnnData object was then converted into a

SingleCellExperiment object for further processing in R. To make this dataset usable with scPred, we continued principal component analysis in the Bioconductor pipeline, and lastly converted the analyzed SCE object to a Seurat object.

Pretrained learning models for basic immune cells

Training and testing of classifiers was performed using the package's inbuilt functions (see above). We used multiple datasets to train and test our package's inbuilt classifiers. In particular, the Sade-Feldman and the Jerby-Arnon scRNA-seq melanoma datasets were used to train and test classifiers for B cells, plasma cells, NK cells, T cells, monocytes, and dendritic cells. In addition, we used the skin cell atlas to train and test more classifiers, many of them are specific for skin cell types: endothelial cells, vascular/lymphatic endothelial cells, Schwann cells, pericytes, mast cells, keratinocytes, and melanocytes. Meanwhile, the Covid-19 cell atlas was used to train and test additional classifiers for immune cell types: CD4+ T cells, CD8+ T cells, NKT cells, CD56dim NK cells, CD56bright NK cells, plasmacytoid dendritic cells, CD14+ monocytes, CD16+ monocytes, platelets and red blood cells. To have enough training and testing sets, the large datasets were splitted into multiple small datasets based on the tissue types (healthy, eczema, and psoriasis tissues in the skin cell atlas) or based on sample collection centers (Cambridge, Sanger, Newcastle center in the Covid-19 cell atlas). Those different datasets contain overlapping cell types, which we used to cross-check the performance of the trained classifiers. Last but not least, all classifiers for pancreatic cell types (alpha, beta, delta, gamma, acinar, and ductal) were trained on the Muraro and tested on the Baron pancreas dataset. At the time of writing, the package ships with classifiers for 32 cell types.

Benchmarking discrete populations using the pancreas datasets

This benchmark was performed using the 6 pancreas datasets and a 6-fold cross-validation scheme. In each fold, one among six datasets was used for training and the other five for testing. Preprocessed and analyzed objects were converted into dgCMatrix (for scClassify), SingleCellExperiment objects (for clustifyr, SingleR, CHETAH, scmap-cluster and scmap-cell), into expression matrices (for SciBet and SCINA) or into CellDataSet objects (for Garnett).

For scAnnotatR, we used the same set of features in the training of pancreatic alpha, beta, delta, gamma, ductal, and acinar classifiers throughout the six folds. For scClassify classifiers training, we followed their basic pipeline by using limma as feature selection method, pearson as similarity calculation method, and WKNN as the KNN algorithm. As Garnett needs a marker gene list as well as

a training set we used two benchmarking strategies. In the first approach, we applied the same list of marker genes that we used for scAnnotatR on six different training sets throughout six folds, while the later approach took the top 10 differentially expressed genes of the cell groups from the corresponding training set. SCINA uses only marker genes, so we used the same top 10 differentially expressed markers as the second benchmark of Garnett does. For all datasets we used Seurat's FindAllMarkers function to find the top 10 differentially expressed genes. For scPred, the originally preprocessed data was provided as input in the form of an Seurat object as scPred does not support other input objects. Additionally, we evaluated scPred re-processing all datasets using the scTransform Seurat pipeline throughout. The scpred_prediction data slot returned by scPredict function was used as scPred's classification. For all other methods, we used the standard pipeline provided by the authors for training and testing. Cells with ambiguous assignments in the source data were excluded from the training and testing process.

The benchmark assesses three aspects of the cell classification accuracy: 1) the overall accuracy of the classification, 2) how well a specific cell type was recognized, and 3) how often cell types that were not part of the reference were misclassified.

The accuracy was calculated based on the total number of correct assignments and correct unassigned cells over the total number of cells in the datasets (Equation 1). Methods that report intermediate/ambiguous cell assignments (scClassify, CHETAH, and scAnnotatR) were assessed twice. Once only an unambiguous correct assignment was considered as correct and once ambiguous assignments were deemed correct as long as they contained the actual correct assignment.

Equation 1: $accuracy = \frac{(Correctly\ assigned\ cells + correctly\ unassigned\ cells)}{Total\ number\ of\ cells}$

The accuracy of specific cell type identifications was assessed by transforming the classification results into a binary matrix where each cell type is represented as one column and each cell as one row. For each cell we record whether it was classified as that specific cell type (1) or not (0). As we know the correct number of specific cells in the training dataset, we can then calculate the sensitivity and specificity per cell type. Summary statistics are then reported as the average sensitivity and specificity across all *n* classified cell types *i* (Equation 2 and 3).

Equation 2: $dataset\ sensitivity = \sum_{i=1}^{n} \frac{True\ Positive_i}{(True\ Positive_i + False\ Negative_i)} \div n$

Equation 3: $dataset\ specificity\ = \sum_{i=1}^{n} \frac{True\ Negative_{i}}{(True\ Negative_{i} + False\ Positive_{i})} \div n$

Similar to the accuracy, classification tools supporting ambiguous/intermediate cell predictions were evaluated twice for their sensitivity and specificity. When accepting ambiguous assignments as correct, correct intermediate cell assignments increase the sensitivity, but the specificity is not penalized by incorrect ambiguous assignments.

The misclassification of unknown cell types was assessed by testing whether cell types that are not part of the training dataset were classified. This rate was defined as the ratio of cells that remained unclassified divided by the total number of cells that should not have been classified (Equation 4). Here, we refer to this rate as the 'unknown population detection rate'.

Equation 4: $Unknown\ population\ detection\ rate\ = \frac{Correctly\ unassigned\ cells}{Total\ number\ of\ unknown\ cells}$

The ambiguous/intermediate cell assignment does not affect the unknown population rate. Therefore, only one unknown population detection rate is reported for cell classification tools providing ambiguous/intermediate prediction.

In order to ensure a fair comparison between tools regarding the unknown population detection rate, we decided to apply a confidence threshold onto SciBet's prediction. Based on the SciBet's documentation, we treated all predictions with confidence below 0.4 (default value) as 'unassigned'.

Benchmarking closely related populations

This benchmark was performed in a multiple-fold cross-validation scheme. Additionally, the benchmark was performed using two levels of annotations. In level one, the datasets are annotated with a low level of detail of immune cell types while in level two, sub-cell types are annotated. Ten datasets were used in this benchmark: the PBMC 3k dataset analyzed by Seurat v3.1 [19]; the PBMC 500 dataset, which was a subset of the PBMC 3k dataset but preprocessed and analyzed by ILoReg v1.0 [15]; the PBMC dataset by Ding *et al*. [12] containing seven subsets corresponding to seven different sequencing protocols; and the SCP345 PBMC dataset [14]. The latter could not be included in the second level of the benchmark as it lacked detailed cell annotations.

In both benchmarks (level one and two), scAnnotatR used the same feature sets as the in-built classifiers for the respective cell types. For scClassify, classifiers were trained by using limma as feature selection method, pearson as similarity calculation method, and WKNN as the KNN

algorithm. Garnett and SCINA used the top 10 differentially expressed genes found by Seurat's FindAllMarkers function. scPred was further evaluated using two additional approaches: once where training and testing datasets were processed by the same pipeline, and once where additionally all classifiers were optimized manually. The training and optimization process was run with all scPred default parameters. The scpred_prediction data slot returned by scPredict function was used as scPred's classification. For all other methods, we used the standard pipeline provided by the authors for training and testing.

Cell types were classified in each dataset and the general accuracy, sensitivity and specificity calculated per cell type as described above. Results are reported as average across all cell types per dataset. For each dataset, we further assessed the unknown population detection rate (see above).

Several tools organise cell types in a hierarchical structure. For scAnnotatR and Garnett, the hierarchical structure of cell types was defined by the user during the training process (scAnnotatR) or when defining markers (Garnett). In level two of the benchmark, these two tools classify major/parent cell types (T cell, monocytes) before classifying their subtypes (CD4+ T cells/CD8+ cells, CD14+ monocytes/CD16+ monocytes). In this case, cells correctly assigned by major cell types (only one predicted cell type per cell) but not classified by any of the child classifiers are also considered to be correct classification.

Runtime estimation

We assessed the runtime on five specifically selected large datasets: the joint Ding PBMC dataset [12], the Reynolds skin cell atlas [4], the Stephenson Covid-19 cell atlas [5], the Zilionis lung dataset [22], and the HIV1 dataset [21] ranging from 31,021 to 647,366 cells. The Sade-Feldman melanoma dataset was used for training. For Garnett and SCINA, top 10 differentially expressed genes of the cell groups from the Sade-Feldman melanoma dataset were provided. For scAnnotatR, all package in-built models were applied. For scClassify classifier training, we tested both non-ensemble learning and ensemble learning. Since the resulting prediction remained the same, we used the non-ensemble learning model in the runtime benchmark. For tools with separate training and predicting processes, we recorded only the time consumed by the prediction. For the other tools, runtime consumed by the main classification functions was recorded, even though those functions might integrate internal training processes. The prediction runtime was recorded as wall clock time. The exact moment of start and end were retrieved using the sys.time() function in the R base package. For tools where training and classification processes are independent, only the

classification running time was measured. All benchmarks were done on an Ubuntu machine with 200GB RAM and 32 cores.

Supplementary Figure 1

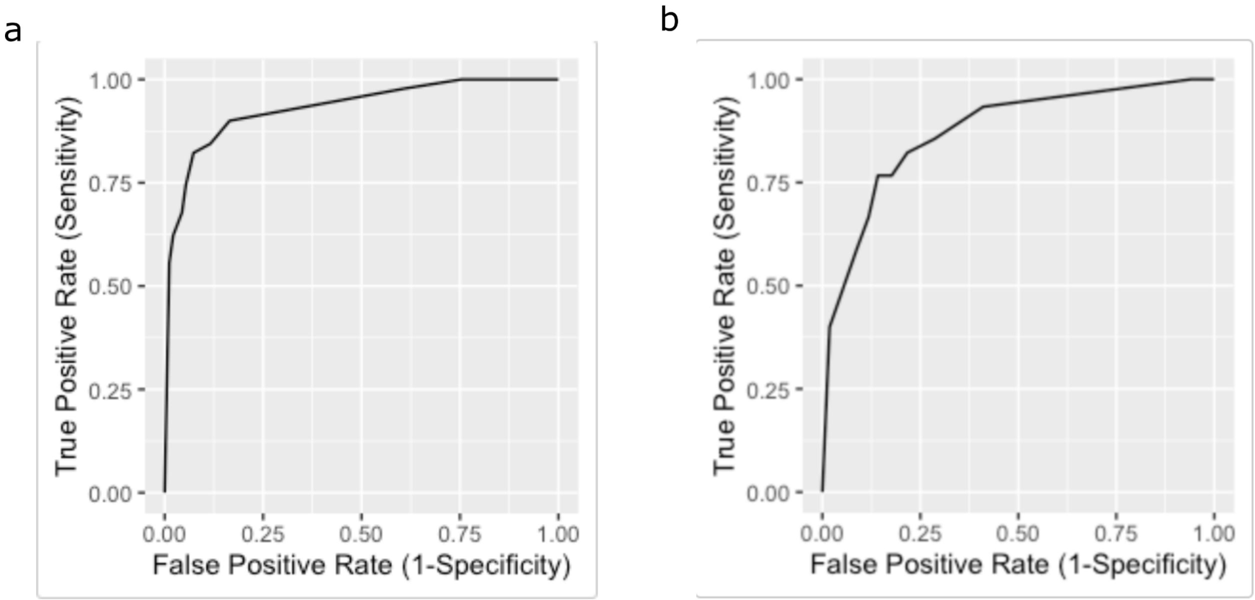

Comparison of the performance of B cell classifiers trained using (a) linear kernel SVM and (b) radial basis function (RBF) kernel SVM. The models were trained on the same training set and test set (on the first 5000 cells of the Zilionis Lung dataset), using the same set of marker genes as the package default models, and all the same core parameters, except that only the sigma was required and used in the RBF SVM, and it was defined as:

*sigma = 1 / (n_features * var(X))*,

while X was the expression matrix and n_features the number of features used.

The linear SVM trained model has better performance than the RBF SVM-based model. In particular, the area under the roc curve (AUC) of the linear SVM model was 0.932, while the AUC of the RBF SVM model was 0.879.

Supplementary Figure 2

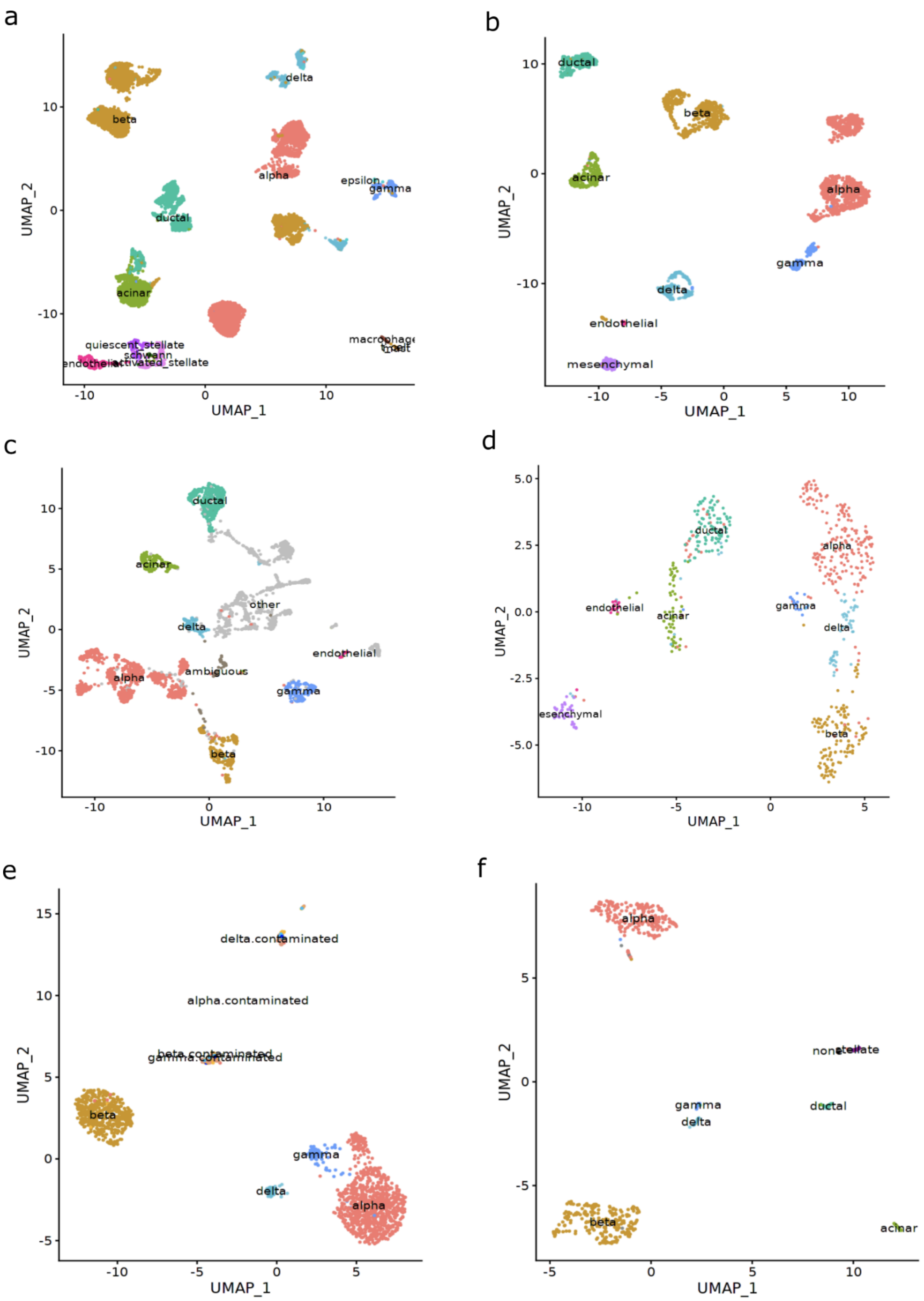

Cell type annotation in pancreas datasets: (**a**) Baron *et al.*, (**b**) Muraro *et al.*, (**c**) Segerstolpe *et al.*, (**d**) Wang *et al.*, (**e**) Xin *et al.*, (**f**) Lawlor *et al.*

Supplementary Figure 3

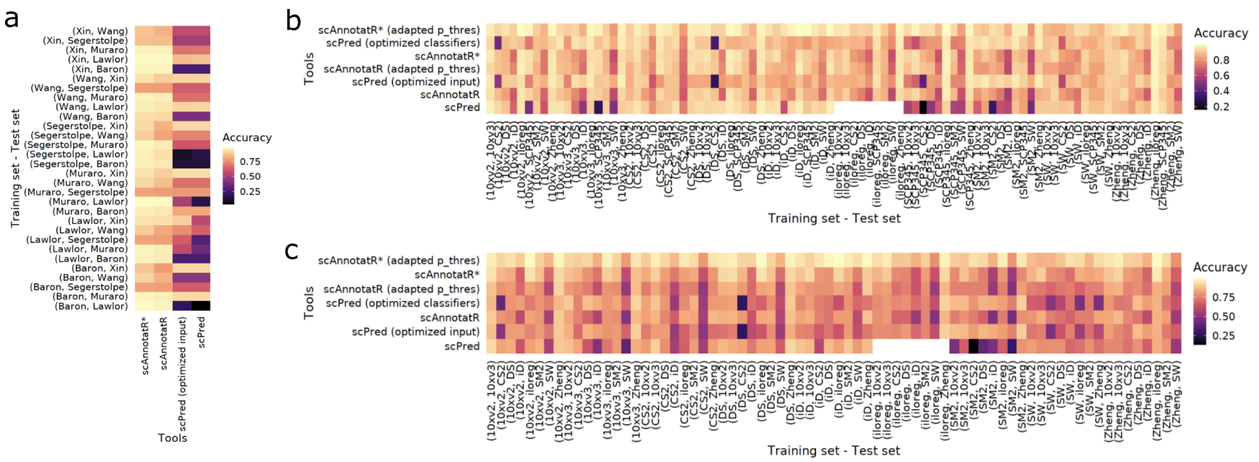

Prediction accuracy of AnnotatR and scPred in the a) pancreas, b) PBMC level 1, and c) PBMC level 2 benchmark. scAnnotatR was evaluated with a fixed prediction probability threshold (0.5) and with adapted prediction thresholds. The (*) after scAnnotatR name refers to the intermediate/ambiguous assignment accepted case. scPred was evaluated in three case: with standard input (processed similarly as other tools' input), with optimized input (all training and testing datasets were processed using the same pipeline), and with optimized input and optimized classifiers (all classifiers were retrained by mixture discriminant analysis (mda)). In the pancreas benchmark, all default scPred classifiers already had good performance. Therefore, further optimization was not possible in this case.

References

1. Langerhans Cells – The Macrophage in Dendritic Cell Clothing. Trends Immunol. 2017;38: 817–828.

2. Sade-Feldman M, Yizhak K, Bjorgaard SL, Ray JP, de Boer CG, Jenkins RW, et al. Defining T Cell States Associated with Response to Checkpoint Immunotherapy in Melanoma. Cell. 2018;175: 998–1013.e20.

3. Jerby-Arnon L, Shah P, Cuoco MS, Rodman C, Su MJ, Melms JC, et al. A Cancer Cell Program Promotes T Cell Exclusion and Resistance to Checkpoint Blockade. Cell. 2018;175. doi:10.1016/j.cell.2018.09.006

4. Reynolds G, Vegh P, Fletcher J, Poyner EFM, Stephenson E, Goh I, et al. Developmental cell programs are co-opted in inflammatory skin disease. Science. 2021;371. doi:10.1126/science.aba6500

5. Stephenson E, Reynolds G, Botting RA, Calero-Nieto FJ, Morgan MD, Tuong ZK, et al. Single-cell multi-omics analysis of the immune response in COVID-19. Nat Med. 2021;27: 904–916.

6. Baron M, Veres A, Wolock SL, Faust AL, Gaujoux R, Vetere A, et al. A Single-Cell Transcriptomic Map of the Human and Mouse Pancreas Reveals Inter- and Intra-cell Population Structure. Cell systems. 2016;3. doi:10.1016/j.cels.2016.08.011

7. Muraro MJ, Dharmadhikari G, Grün D, Groen N, Dielen T, Jansen E, et al. A Single-Cell Transcriptome Atlas of the Human Pancreas. Cell systems. 2016;3. doi:10.1016/j.cels.2016.09.002

8. Segerstolpe Å, Palasantza A, Eliasson P, Andersson E-M, Andréasson A-C, Sun X, et al. Single-Cell Transcriptome Profiling of Human Pancreatic Islets in Health and Type 2 Diabetes. Cell Metab. 2016;24: 593.

9. Wang YJ, Schug J, Won KJ, Liu C, Naji A, Avrahami D, et al. Single-Cell Transcriptomics of the Human Endocrine Pancreas. Diabetes. 2016;65. doi:10.2337/db16-0405

10. Xin Y, Kim J, Okamoto H, Ni M, Wei Y, Adler C, et al. RNA Sequencing of Single Human Islet Cells Reveals Type 2 Diabetes Genes. Cell Metab. 2016;24. doi:10.1016/j.cmet.2016.08.018

11. Lawlor N, George J, Bolisetty M, Kursawe R, Sun L, Sivakamasundari V, et al. Single-cell transcriptomes identify human islet cell signatures and reveal cell-type-specific expression changes in type 2 diabetes. Genome Res. 2017;27: 208–222.

12. Ding J, Adiconis X, Simmons SK, Kowalczyk MS, Hession CC, Marjanovic ND, et al. Systematic comparative analysis of single cell RNA-sequencing methods. Cold Spring Harbor Laboratory. 2019. p. 632216. doi:10.1101/632216

13. Zheng GXY, Terry JM, Belgrader P, Ryvkin P, Bent ZW, Wilson R, et al. Massively parallel digital transcriptional profiling of single cells. Nat Commun. 2017;8: 14049.

14. Single Cell Portal. [cited 1 Jul 2021]. Available: https://singlecell.broadinstitute.org/single_cell/study/SCP345/ica-blood-mononuclear-cells-2

-donors-2-sites

15. Smolander J. ILoReg package manual. 27 Oct 2020 [cited 7 Dec 2020]. Available: https://bioconductor.org/packages/release/bioc/vignettes/ILoReg/inst/doc/ILoReg.html

16. Pancreas. [cited 18 Nov 2020]. Available: https://hemberg-lab.github.io/scRNA.seq.datasets/human/pancreas/

17. Risso D, Lun* A. Overview of the scRNAseq dataset collection. [cited 8 Dec 2020]. Available: http://bioconductor.org/packages/release/data/experiment/vignettes/scRNAseq/inst/doc/scRNAseq.html

18. Chapter 32 Lawlor human pancreas (SMARTer). [cited 28 Jun 2021]. Available: https://github.com/Bioconductor/OrchestratingSingleCellAnalysis

19. Satija Lab. [cited 23 Nov 2020]. Available: https://satijalab.org/seurat/v3.1/pbmc3k_tutorial.html

20. Single Cell Portal. [cited 7 Dec 2020]. Available: https://singlecell.broadinstitute.org/single_cell/study/SCP424/single-cell-comparison-pbmc-data#/

21. Single Cell Portal. [cited 8 Dec 2020]. Available: https://singlecell.broadinstitute.org/single_cell/study/SCP256/integrated-single-cell-analysis-of-multicellular-immune-dynamics-during-hyper-acute-hiv-1-infection

22. Zilionis R, Engblom C, Pfirschke C, Savova V, Zemmour D, Saatcioglu HD, et al. Single-Cell Transcriptomics of Human and Mouse Lung Cancers Reveals Conserved Myeloid Populations across Individuals and Species. Immunity. 2019;50: 1317–1334.e10.

23. Chapter 42 HCA human bone marrow (10X Genomics). [cited 28 Jun 2021]. Available: https://github.com/Bioconductor/OrchestratingSingleCellAnalysis

24. Goh I. Development Cell Atlas. [cited 23 Sep 2021]. Available: https://developmentcellatlas.ncl.ac.uk/datasets/hca_skin_portal/

25. COVID-19 Cell Atlas. [cited 23 Sep 2021]. Available: https://covid19cellatlas.org/
